# Spatio-temporal aspects of the interplay of cancer and the immune system

**DOI:** 10.1007/s10867-019-09535-3

**Published:** 2019-11-26

**Authors:** Vladimir P. Zhdanov

**Affiliations:** 1 Section of Biological Physics, Department of Physics, Chalmers University of Technology, Göteborg, Sweden; 2 Boreskov Institute of Catalysis, Russian Academy of Sciences, Novosibirsk, Russia

**Keywords:** Cancer, Tumour, Growth, Diffusion, Kinetic model

## Abstract

The conventional mean-field kinetic models describing the interplay of cancer and the immune system are temporal and predict exponential growth or elimination of the population of tumour cells provided their number is small and their effect on the immune system is negligible. More complex kinetics are associated with non-linear features of the response of the immune system. The generic model presented in this communication takes into account that the rates of the birth and death of tumour cells inside a tumour spheroid can significantly depend on the radial coordinate due to diffusion limitations in the supply of nutrients and/or transport of the species (cells and proteins) belonging to the immune system. In this case, non-trivial kinetic regimes are shown to be possible even without appreciable perturbation of the immune system.

Cancer occurs via initiation, tumour growth, and propagation of metastases. During these stages, its development depends on the response of the immune system (reviewed in [1, 2]) and the enhancement of this response can be used as a basis for efficient anticancer therapies [3]. The interplay of cancer and the immune system is complex and our understanding of this interplay remains limited. At the conceptual level, this interplay can be illustrated by using the corresponding kinetic models. Customarily, such models are temporal and operate with the population of cancer cells forming a tumour and populations of the species (cells and proteins) belonging to the immune system (reviewed in [4, 5]; see also recent treatment [6]; for a more general perspective on the kinetic models of cancer, see reviews [7–10]). In this framework, the growth or elimination of the population of tumour cells is predicted to be described by the first-order equation and to be exponential provided their number is small and their effect on the immune system is negligible, and accordingly the emphasis is shifted towards the complexity related to non-linear features of the behaviour of the immune system. The effect of the immune system on the initial growth of tumour cells was also theoretically analyzed [11] in the contexts of the experiments determining the lifetime risk of cancer (Ref. [12]; briefly reviewed in [13]) and the interaction of tumours via the immune system. The spatio-temporal aspects were, however, not treated there in detail. In some of the recent multivariable models (see, e.g. [14–16]), these aspects are taken into account to some extent, but the corresponding mathematical analysis is rather cumbersome and the results reported do not allow one to see the physics behind. Herein, I (i) propose a generic spatio-temporal model focused on the initial phase of the growth of a tumour in the regime where its effect on the the populations of species belonging to the immune system is negligible and (ii) show that even in this limit the kinetics are not necessarily reduced to the exponential growth or elimination of the population of cancer cells.

To illustrate the general introduction above and to articulate the novelty of my report, I recall one of the conventional temporal models ([17]; reviewed in detail in [4, 5]) and show what it predicts in the situation when the effect of tumour cells on the the populations of species belonging to the immune system is negligible. The corresponding equations for the populations of tumour cells (*n*_t_), effector cells (*n*_e_) and interleukin-2 (*n*_p_) are as follows [4, 17]:

1 $$ \frac{dn_{\mathrm{t}}}{dt}= k n_{\mathrm{t}}(1-n_{\mathrm{t}}/n_{*}) - \gamma n_{\mathrm{e}} \frac{n_{\mathrm{t}}}{m_{1}+n_{\mathrm{t}}}, $$

2 $$ \frac{dn_{\mathrm{e}}}{dt}= w_{\mathrm{e}}-\kappa_{\mathrm{e}}n_{\mathrm{e}} +v_{1} n_{\mathrm{t}} +v_{2} n_{\mathrm{e}} \frac{n_{\mathrm{p}}}{m_{2}+n_{\mathrm{p}}}, $$

3 $$ \frac{dn_{\mathrm{p}}}{dt}= w_{\mathrm{p}}-\kappa_{\mathrm{p}}n_{\mathrm{p}} +v_{3} n_{\mathrm{e}} \frac{n_{\mathrm{t}}}{m_{3}+n_{\mathrm{t}}}, $$

where *k*, *w*_e_, *w*_p_, *κ*_e_ and *κ*_p_ are the birth and death rate constants and rates, *n*_∗_ is the maximum population of tumour cells, *γ* is the rate constant of elimination of tumour cells and *v*_1_, *v*_2_, *v*_3_, *m*_1_, *m*_2_ and *m*_3_ are the other rate constants and parameters characterizing the function of the immune system. The analysis of Eqs. 1–3 can be simplified taking into account that on the time scale of the tumour growth the response of the immune system is rapid, and accordingly (2) and (3) can be solved in the steady-state approximation by setting *dn*_e_/*dt* = *dn*_p_/*dt* = 0. Describing the initial phase of the growth of a tumour, one can in addition neglect the effect of tumour cells on the immune system. Mathematically, this means that the third term on the right-hand part in Eq. 3 can be neglected, and accordingly one has:

4 $$ n_{\mathrm{p}}=w_{\mathrm{p}}/\kappa_{\mathrm{p}}  \text{and}  n_{\mathrm{e}}= \frac{w_{\mathrm{e}}}{\kappa_{\mathrm{e}} - v_{2}w_{\mathrm{p}}/(m_{2}\kappa_{\mathrm{p}} +w_{\mathrm{p}})}. $$

Along this line, one can neglect *n*_t_/*n*_∗_ in the first term and *n*_t_ in the denominator of the second term in Eq. 1. Then, using expression (4) for *n*_e_, Eq. 1 can be rewritten as:

5 $$ \frac{dn_{\mathrm{t}}}{dt}= \left (k - \frac{\gamma w_{\mathrm{e}}} {m_{1}[\kappa_{\mathrm{e}} - v_{2}w_{\mathrm{p}}/(m_{2}\kappa_{\mathrm{p}} +w_{\mathrm{p}})]} \right ) n_{\mathrm{t}}. $$

This first-order equation predicts exponential growth or elimination of the population of tumour cells depending on the sign of the combination of the parameters in the parentheses. More complex kinetic features including non-trivial steady states are also possible in this model but only provided the population of tumour cells is appreciable and the immune system operates in a non-linear regime (i.e., provided the third term on the right-hand side in Eq. 3 is not negligible).

In the model I use, the evolution of a tumour is described as:

6 $$ \frac{dn_{\mathrm{t}}}{dt}= W_{\mathrm{b}} - W_{\mathrm{d}}, $$

where *n*_t_ is the number of cells forming a tumour, and *W*_b_ and *W*_d_ are the rates of birth and death of these cells. To calculate these rates, the tumour is assumed to be spherical with radius *R*, and accordingly the population of tumour cells is expressed via its volume as:

7 $$ n_{\mathrm{t}}=4\pi R^{3}/3 v, $$

where *v* is the volume per cell.

The growth of the population of tumour cells is considered to be determined by the nutrient distribution inside a tumour spheroid. The corresponding equation for the nutrient concentration at *r* ≤ *R* (*r* is the radial coordinate) is represented as:

8 $$ \frac{\partial c}{\partial t} = D \frac{1}{r^{2}} \frac{\partial }{\partial r} \left (r^{2} \frac{\partial c}{\partial r} \right ) - \eta c, $$

where *D* is the nutrient diffusion coefficient, and *η* is the rate constant associated with the nutrient consumption by tumour cells. The boundary condition for this equation is:

9 $$ c(R)=c_{\circ}, $$

where *c*_∘_ is the nutrient concentration outside the tumour. Taking into account that on the time scale of the tumour growth the nutrient diffusion inside the tumour is rapid, Eq. 8 can be solved in the steady-state approximation by setting *∂c*/*∂t* = 0. The corresponding textbook solution of Eq. 8 is given by:

10 $$ c(r) = \frac{R \sinh (r/\lambda )}{r \sinh (R /\lambda )} c_{\circ}, $$

where *λ* ≡ (*D*/*η*)^1/2^.

The local birth rate of tumour cells can be considered to be proportional to *c*(*r*). In this approximation, the total birth rate can be represented as:

11 $$ W_{\mathrm{b}} = k n_{\mathrm{t}} F(R/\lambda ), $$

where *k* is the birth rate constant (*c*_∘_ is considered to be included into this rate constant), *kn*_t_ is the birth rate calculated assuming the diffusion limitations to be negligible, and

12 $$ F(R/\lambda ) = {{\int}_{0}^{R}} c(r) 4\pi r^{2} dr\left / \left (\frac{4\pi R^{3}}{3} c_{\circ}\right ) \right . = \frac{3\lambda}{R} \left (\coth (R/\lambda) - \frac{\lambda}{R}\right ) $$

is the factor taking the diffusion limitations into account. This factor is represented above as a function of *R*/*λ* for the convenience of its derivation as it is usually done in the literature focused on the kinetics of catalytic reactions occurring inside grains (the corresponding models of catalytic reactions can be tracked down to the seminal study by Thiele [18]). In the context of tumour growth, *R* should be expressed via *n*_t_ taking relation (7) into account, i.e., Eq. 11 should be rewritten as:

13 $$ W_{\mathrm{b}} = k n_{\mathrm{t}} F(n_{\mathrm{t}}^{1/3}/\chi ), $$

where *χ* ≡ (4*π*/3*v*)^1/3^*λ*.

The elimination of tumour cells is considered controlled by the species (cells or proteins) belonging to the immune system. Mathematically, this process can be described in analogy with Eqs. 8–10 by replacing *c*, *D*, *η* and *λ* by the corresponding parameters, *c*_∗_, *D*_∗_, *η*_∗_ and *λ*_∗_≡ (*D*_∗_/*η*_∗_)^1/2^. Then, by analogy with Eq. 13, the death rate is represented as:

14 $$ W_{\mathrm{d}} = \gamma n_{\mathrm{t}} F(n_{\mathrm{t}}^{1/3}/\chi_{*} ), $$

where where *γ* is the death rate constant, and *χ*_∗_≡ (4*π*/3*v*)^1/3^*λ*_∗_.

If the population of tumour cells is small ($n_{\mathrm {t}}^{1/3} \ll \min \limits (\chi , \chi _{*})$), the diffusion limitations are negligible, i.e. $F\simeq 1$, $W_{\mathrm {b}} \simeq k n_{\mathrm {t}}$, and $W_{\mathrm {d}} \simeq \kappa n_{\mathrm {t}}$, and accordingly (6) can be simplified as:

15 $$ \frac{dn_{\mathrm{t}}}{dt}= (k-\gamma ) n_{\mathrm{t}}. $$

This equation predicts exponential growth or elimination of the population of tumour cells at *k* > *γ* and *k* < *γ*, respectively.

If the population of tumour cells is larger ($n_{\mathrm {t}}^{1/3} \gg \min \limits (\chi , \chi _{*})$), the birth and death rates of tumour cells are controlled by diffusion, i.e. $F(n_{\mathrm {t}}^{1/3}/\chi )\simeq 3\chi /n_{\mathrm {t}}^{1/3}$, $F(n_{\mathrm {t}}^{1/3}/\chi _{*} )\simeq 3\chi _{*}/n_{\mathrm {t}}^{1/3}$, $W_{\mathrm {b}} \simeq 3 \chi k n_{\mathrm {t}}^{2/3}$ and $W_{\mathrm {d}} \simeq 3 \chi _{*} \gamma n_{\mathrm {t}}^{2/3}$, and accordingly (6) can be simplified as:

16 $$ \frac{dn_{\mathrm{t}}}{dt}= 3(\chi k-\chi_{*}\gamma ) n_{\mathrm{t}}^{2/3}. $$

This equation predicts growth or elimination of the population of tumour cells at *χk* > *χ*_∗_*γ* and *χk* < *χ*_∗_*γ*, respectively. It is of interest that the growth is algebraic rather than exponential.

Comparing (15) and (16), one can conclude that the growth or death of the population of tumour cells is predicted in both cases provided $k >\max \limits (\gamma , \gamma \chi _{*}/\chi )$ and $k <\min \limits (\gamma , \gamma \chi _{*}/\chi )$, respectively. In other words, the model predicts unlimited growth or extinction in this case. For $\min \limits (\gamma , \gamma \chi _{*}/\chi )< k< \max \limits (\gamma , \gamma \chi _{*}/\chi )$, the situation is less trivial because there is a stable or unstable steady state depending on whether *χ*_∗_/*χ* is larger or smaller than unity as illustrated graphically in Fig. 1.

**Fig. 1:**
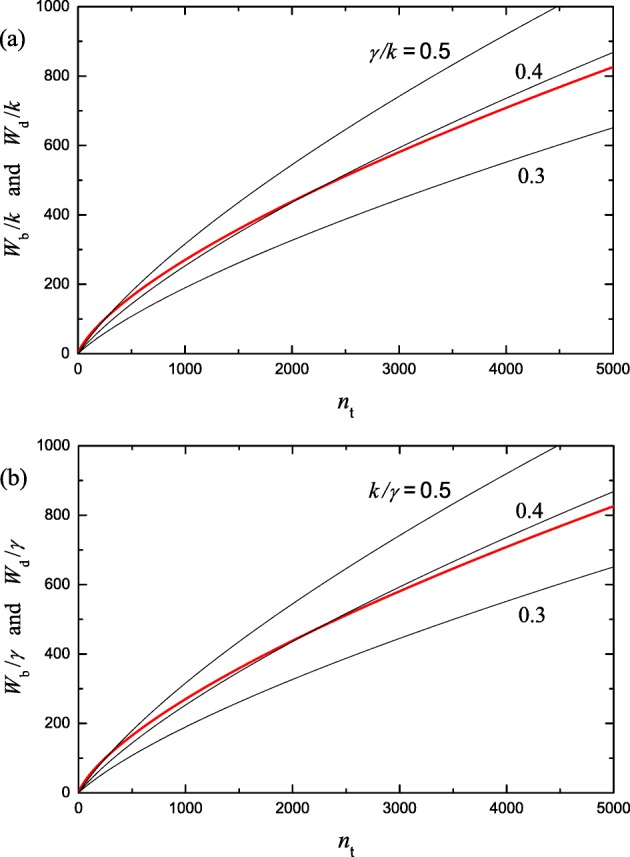
Normalized birth and death rates of tumour cells [(13) and (14)] as a function of their number. **a** The thick line shows the birth rate for *χ* = 1. The thin lines represent the death rate for *χ*_∗_ = 3 and *γ*/*k* = 0.3, 0.4 and 0.5. In this case, the model predicts unlimited growth for *γ*/*k* = 0.3 and existence of a stable steady state for *γ*/*k* = 0.4 and 0.5. **b** The same curves are used to illustrate the existence of a stable steady state. In this case, the thick line shows the death rate for *χ*_∗_ = 1, whereas the thin lines represent the birth rate for *χ* = 3 and *k*/*γ* = 0.3, 0.4 and 0.5. Under these conditions, the model predicts extinction for *k*/*γ* = 0.3 and existence of a unstable steady state for *k*/*γ* = 0.4 and 0.5

Thus, the model clearly shows that, with inclusion of spatial features, non-trivial kinetic regimes of tumour growth may be possible even at relatively small populations of tumour cells in the situations when the effect of tumour cells on the population of the species (cells and proteins) belonging to the immune system is negligible. Physically, this is related to the transition from the kinetically limited birth and death to diffusion-limited birth and death with increasing population of tumour cells. For birth and death, this transition can easily take place at different populations of cells, and it can result in the appearance of a non-trivial stable or unstable steady state.

Finally, I can add that the model under consideration can be extended in different directions. For example, its current version implies that the tumour cells are of one type. In reality, the population of tumour cells is well known to be heterogeneous [19, 20], and this factor can easily be taken into account in the analysis presented. The model can also be reformulated in the terms of chemotherapy, and accordingly it can be used in the latter area as well.
